# Dr. Alder's Proofs of His Theory from the Remote Causes of Diseases and Fevers

**Published:** 1810-04

**Authors:** Thomas Alder


### Dr. Alder's *Proofs of his Theory from the remote Causes of Diseases and Fevers.*

( In Continuation. )

In the early part of these communicaitons, I took tolerably full notice of the sources and prevalence of bad air; but this was done with a view to shew the application of this in cases where "contagion" was supposed to be the chief or sole agent in the production of diseases, and to point out the great similarity (I may say *identity)* in certain fevers, obviously owing to bad air, and almost generally or altogether attributed to it; and others, in which the application of bad air is not so evident, and which (partly from that circumstance, and partly from their prevailing more extensively) have, till within these few years, nearly universally, and now are very generally, particularly in Europe, attributed to contagion. It was written also with the view of shewing how destructive it would be to our commerce, and consequently to the kingdom, to enforce quarantines under many circumstances which have happened, and are likely to occur again in such a way as our present most prevailing febrile-contagion doctrines require it to be enforced; or in any manner indeed, which at all approaches to such a way: but my subject now requires me to write with a more undivided and definite object; and I have something more to add.

I must first refer the reader to what I have there written, and then I will ask him, if the following abstract of some of the more interesting articles in Mr. Webster's invaluable History of Epidemics, do not support most of my chief positions?

I do not know that my readers will at first clearly perceive what the meteors, droughts, &c. there mentioned prove; but I will point out what they seem to me to demonstrate, either as I go along or afterwards; and I shall at the same time offer such other observations as shall seem demanded by my present subject. I shall begin with Mr. Webtser's history, at the year before the Christian æra, 433.

B. C. 433, 4, 5. Great heat of the earth preceded the plague Thucidydes describes. Earthquakes attended it, preceded, and followed it. Violent ones followed it for two years, and extended all over the earth. The plague too was very general; but it was worse at Athens, from the people being there crowded up in hot weather, and desponding. Rome had had a plague a year or two before; but it was subject to plagues, owing, no doubt, to the marshes marshes and mephitic caverns near it, and to the cause of the many earthquakes which happened near it.

*After the Christian Æra*, 165, 6, 7; and more or less till 173, 5, 8, and afterwards. During all this period there was an immense mortality over the face of the then known world, with many earthquakes in various places, much putrefaction and unwholsomeness in the seasons, and often famine. They happened in a great measure in the time of Marcus Aurelius and Lucius Vera; and are mentioned by the learned Dr. Arbuthnot, and a late writer (as well as myself) as destroying half the human race in the time of *Marc Anthony*; Dr. Arbuthnot led me into the error; Marcus Aurelius was called Marcus Antoninus. It is computed they destroyed more than half of the human race.

From 250, at intervals, till 263 or 266. For about seven years before this, the earth was agitated by most violent earthquakes; and about two years before, the sea rose to a most unusual height on the Eastern coast of this kingdom, and overflowing some hundred thousand acres in the county of Lincoln, all of which, at this day, have scarcely been quite recovered. Earthquakes too followed the plagues; an eruption of mount Ætna preceded, and an eruption of mount Vesuvius followed them. The plagues which prevailed in these times destroyed about half the human race. They were most fatal at Rome in 252 and 263, and in Scotland in 266. The air in some places was remarkably thick and impure, causing great darkness, and a deposition of putrescent dew.

407, 8, et seq. Prodigious meteors, violent and numerous earthquakes; heat of the earth, which destroyed vegetation, and caused extensive and severe famine. Then pestilence, which, according to Nicephorus, destroyed almost all Europe, and a considerable part of Asia and Africa.

In 445, 6, 7, 8, 9. Tremendous earthquakes for many months, over a great part of the globe. At Constantinople, in particular, very early, severe, and long, and often repeated. Dreadful plagues, not only there, but universally, and of several years duration. Very severe in England in 448 and 9.

From 543, during 50 years, the plague travelled all over the world, sometimes going progressively, sometimes making a circuit. But much worse at some times, and in some places, than in others. Procopius says, it did not spread *by contact*, and on that account people were emboldened to wait upon the sick, and bury the dead. Yet it attacked people of all ages and descriptions, and in all situations, and almost depopulated the world. It often travelled *in a*

*very*

*very narrow compass,* (according to Evagreus) *leaving many places untouched,* afflicting others not far off very severely. At first it passed slightly over several cities, *it attacked the half of others,* in some only a few houses, but in others it scarcely left an inhabitant.

From this time to the year 1000, there were seven or eight general plagues, with their usual attendants, earthquakes, volcanic eruptions, unwholesome seasons, &c.

In 1000 we have the first account of the eruption of Mount Heckla. It emitted two meteors like globes of fire. These were followed by severe earthquakes in England, by a very cold winter, and in summer by fluxes, epidemics, and mortal fevers.

In 1004 an eruption of Heckla and a violent earthquake. In 1005, earthquakes in Italy for three months. Then a famine and plague over the whole earth for three years, which, according to contemporary authors, appeared to destroy more than one half of the human race. Afterwards Vesuvius poured out immense quantities of lava.

In 1174 we find the first epidemic catarrh mentioned.

In 1196, in England a universal and very fatal epidemic prevailed, called a burning ague; it came on after heavy rains, which caused a dearth, and with an earthquake and singular fiery appearances in the sky. A similar ague had been epidemic here before.

In 1274, the rot in sheep was first taken notice of in England.

In 1346, or a little earlier, a fœtid destructive vapour is said to have broken out of the earth in Cathay in China, which caused a most extensive plague. It is certain this plague appeared in 1346, in Egypt, Syria, Turkey, and Greece; and in 1347 in Sicily, Pisa, Genoa, and other parts of Italy; and in 1348 in the South of France, first at *Avignon* (which is at a distance from the sea), then in the other parts of the kingdom, and in the southern provinces of Spain. At the close of the same year it made its appearance in England, first in Dorsetshire, and soon travelled all over the kingdom. In 1349 it over-run Ireland, Holland and Scotland; and in 1350, all Germany, Hungary, and the North of Europe. It raged in winter as well as in summer, even in the North of Europe. It was preceded and attended by many unusual phenomena. In 1347 a frightful comet appeared. During the prevalence of the disease the whole earth was shaken by most tremendous earthquakes. All Germany was shaken in 1346. In 1349 Italy was shaken, and Sicily to its foundation. In Greece many cities were overthrown. Over Avignon in France, a pillar of fire wa seen for an hour; bnt the seasons after were very moist. It was very fatal in Greenland and Iceland; also at Avignon, Montpellier, and Marseilles, especially among the monks; the Clergy in England too suffered much. A bad typhoid pneumonia is said to have prevailed epidemically with this, but it probably was no more than a modification of the other. Cattle, fowls, and even fishes were affected. Brabant and Milan escaped this pestilence, and so did several high places.

In 1354 an epidemic madness prevailed in England.

In 1361 a general plague prevailed; it was unlike almost all others, as it was as violent in country houses as in towns, and was most violent in *mountainous districts.* In 1373 an epidemic madnes reigned among the lower order of people in England. In 1374 it prevailed in France and Italy.

In 1485, the sweating plague in England seized Englishmen first and *chiefly*; but it attacked people of *all* countries, and in *most* of them. The *common* plague was epidemic in Italy and Germany *previous* to it, and Denmark *while it prevailed.*

In 1490 an epidemic leprosy, causing ulcers from head to foot, prevailed in Germany, and an epidemic elephantiasis had, long before, reigned in Italy.

In 1510, after an eruption of Mount Heckla, a catarrh spread universally through Europe.

In 1515, a malignant catarrh or pneumonia, ravaged Holland, and often killed people in a single day.

In the spring, 1517, a similar disease was epidemic in England; *and this by Midsummer became the sweating sickness.* These two are said to have destroyed half the people in England. In 1540, a pestilential ague and a dysentery were epidemic and mortal in England.

In 1557, fevers were epidemic generally. In 1558 an epidemic dysentery prevailed in France; and epidemics and semitertians in Holland.

In 1576, while an epidemic reigned (among other places) in Verona and Padua very severely, Vicenza, *which lies between them,* escaped, and was not attacked till the following year.

In 1580, a very fatal catarrh seized Sicily, and in a few months spread all over Europe.

In 1591, one of a similar kind prevailed as universally, *and was the immediate forerunner of the plague.*

The year 1600 was remarkable for a pestilence in almost every part of Europe, which continued for two or three years, years, and caused great mortality. Subterraneous fires, and famine, prevailed at the same time.

In 1610 there was an epidemic catarrh, which the Spaniards say was the first that visited them.

In 1618 and 1619, the yellow fever made great ravages among the American Indians. In the following years it attacked the Indians also, and the new settlers, and since then often.

From 1620 to 1660, the plague and other epidemic diseases, (with earthquakes and volcanic eruptions, &c.) were frequent and fatal all over Europe and in America. In Europe they began in Hungary, and spread *along the Danube*, and afterwards *along the Rhine*. The same continued (though with less violence, except eruptions from Mount Etna, which lasted for years) *till many years after the plague in London in* 1665 *and* 6. Of what happened in Africa and Asia we have little account. In 1663, there was in England, a memorable mortality among cattle and sheep; *the liver, and sometimes the lungs, having worms.* In 1664 many cattle died. In 1665 the plague appeared in Holland and England. In London, *pneumonia was epidemic in winter, having succeeded bad epidemic tertians. A pestilential fever raged in the spring; and the plague at Midsummer. As it subsided, the dysentery and other diseases were again epidemic.* From all this it is clear, that one great cause, operating differently, according to seasons, &c. produced all these complaints, both in cattle and in men.

In 1682, a mortal disease spread among the cattle in Italy, Switzerland, and Germany, called Angina Maligna. Cattle died of it in twenty-four hours. In Germany and Poland it often travelled very slow, sometimes not more than *half a mile in an hour*: so that it often took as much time to travel *ten* miles, as the epidemic influenza which attacks the human species does to go *one hundred* or many more. The slowness of its progress shews either that the cause of it was heavy and confined near the ground, when the current of air could affect it but little; or that the air was calm, *or both*; which last is most probable, as it is not certain that it arose altogether from the ground.

On the 10th of January, 1693, a most terrible earthquake shook Sicily and Naples. The sky from serene became obscured with a vapour of a *reddish or yellow hue.* Malignant fevers, small-pox, madness, dullness, sottishness, and melancholy with delirium and lethargy immediately followed. Another earthquake happened a little before this; and another occurred in 1693, in Banda, in the the Indian Sea; and after both, *a sulphureous smell* was perceived. In 1695, *apoplexy was epidemic in Italy.*

From 1702 to 1711, the plague prevailed in many parts of the north of Europe, after, and partly at the same time with the plague in the Levant, and the bilious fever, &c. in America. Earthquakes and volcanic eruptions occurred as usual on such occasions. In 1711 also, a very fatal disease raged among the cattle in Italy.

In March, 1719, an immense meteor passed the heavens, illuminating the earth, and bursting with a tremendous report; its diameter was calculated by Dr. Halley at a mile and a half. In Europe, malignant fevers were prevalent, and the mortality great. In 1718, 19, 20, and 21, fatal fevers took off numbers who lived near the slaughter-houses in Cork. In 1719, 20, and 21, the plague prevailed at *Aleppo*, and *(contrary to what is usual)* came from the north. In 1720 and 21, the plague was very destructive at *Marseilles and other places in France*. Earthquakes and volcanic eruptions during this period, and about it, *were frequent*. In 1723, in some districts in America, the burning ague was fatal, but was partly owing to a local cause, viz. *draining swamps.*

In 1729 and 30, *a catarrh marched all over Europe in five months*,—much too short a time to allow it to do so by contagion.

In 1733, a catarrh spread all over Europe, and probably nearly all over the earth. In 1734 and 1735, many diseases were epidemic in Britain, and earthquakes were felt there. In 1736 and 7, an ulcerous sore throat and a malignant pneumonia were epidemic in France. And at the same time a very malignant sore throat travelled in America from East to West. Cold and wet weather greatly increased it. The plague too, about the same time, was very fatal in Egypt. In short, epidemics were remarkable; and earthquakes, volcanic eruptions, and meteors equally so. In 1738, *in England, vertigoes, apoplexies, and sudden deaths were very common.* Oczackow also, Barbadoes, and New Spain had the plague. From 1740 to 1744, pestilential diseases prevailed throughout all the known world. The European catarrh of this period spread over England *in a few weeks*; it came from the South. Earthquakes, volcanic eruptions, and meteors were frequent.

From 1759 to 1762, the plague was very fatal in several places of Europe, Asia, Africa, and America; prevailing with earthquakes, volcanic eruptions, &c. as before. After it declined, dysenteries, and sometimes pleurisies were epidemic in several places for some years. In 1771 and 2, after after great earthquakes, &c. there was a plague at Constantinople, and in Poland, Russia, and other places.

In June 1783, an eruption began from Mount Heckla, and it continued with violence for several months: it was *preceded by a drying-up of the springs, and a great fog over Iceland for many months;* which, at last, spread *over Europe,* and gave great alarm: and not without cause, for the influenza *soon followed; and then other diseases and earthquakes in various parts.* The Nile at this time did not overflow as usual; and the plague was very mortal in Egypt.

At the end of the year 1787, eruptions happened from the mountains Etna and Vesuvius: and early in the year 1788, the influenza broke out in Sicily, Italy, and Germany; and soon reached Poland, Russia, Britain, and France. There was great mortality in the East of Europe.

In the next year *crops failed over the whole Earth;* and in July there was a *tremendous earthquake,* which was principally felt in Iceland. Then *almost all the haddocks in the North Sea died.* In October, *Mount Vesuvius was in a state of eruption* for several weeks. In America there was great *darkness* in Kentucky. The influenza appeared soon after, and then the scarlet fever.

1791, 2, and 3, were pestilential years in many places, preceded by earthquakes, and followed by a great eruption of Mount Vesuvius.

In 1796, several parts of America were sickly; and early in the year following, earthquakes and volcanic eruptions were very tremendous in South America. Soon after, a great mortality was observed *among fish and cats.* Then, in summer, the bilious plague began, and continued in autumn. After that, dysenteries, the influenza, pneumonia, and scarlet fever prevailed, and continued during winter. In 1798, the bilious plague was very general in America early in autumn, or after great rains, and with extremely sultry weather; and an earthquake of considerable extent followed it. But I have not convenient room here to do Mr. Webster full justice. He has mentioned many other important circumstances; and particularly, that the following phænomena were usually joined (some or more of them) with epidemic sickness, as well as earthquakes, meteors, and volcanic eruptions; viz. comets, heat of the earth and dryness; drying-up of the springs; failure of crops; famine; very unusual seasons in other ways, sometimes very moist or wet; thick fogs or darkness for days or weeks; fœtid clouds or dews; much putrefac-

 ion; tion; the generation of many insects; great armies of locusts; the death of cattle, sheep, or cats, &c.; the flight of birds; great sickness and mortality among fish; *looming, &c. in the air*; great swelling of the sea, &c.; and many of these circumstances I find noticed by other authors, more than by Mr. Webster. I shall beg leave to examine how these concomitant circumstances are connected in my next.

I am, &c.

THOMAS ALDER.

( To be Continued. )

